# Comparative and phylogenetic analysis of complete chloroplast genome sequences of *Salvia* regarding its worldwide distribution

**DOI:** 10.1038/s41598-023-41198-y

**Published:** 2023-08-31

**Authors:** Dade Yu, Yifei Pei, Ning Cui, Guiping Zhao, Mengmeng Hou, Yingying Chen, Jialei Chen, Xiwen Li

**Affiliations:** 1 Institute of Chinese Materia Medica, China Academy of Chinese Medical Sciences, Beijing, 100700 China; 2 Shandong Academy of Chinese Medicine, Jinan, 250014 China; 3 College of Traditional Chinese Medicine, Yunnan University of Chinese Medicine, Kunming, 650500 China; 4 College of Pharmacy, Henan University of Chinese Medicine, Zhengzhou, 450046 China

**Keywords:** Plant physiology, Plant evolution

## Abstract

*Salvia* is widely used as medicine, food, and ornamental plants all over the world, with three main distribution centers, the Central and western Asia/Mediterranean (CAM), the East Aisa (EA), and the Central and South America (CASA). Along with its large number of species and world-wide distribution, *Salvia* is paraphyletic with multiple diversity. Chloroplast genomes (CPs) are useful tools for analyzing the phylogeny of plants at lower taxonomic levels. In this study, we reported chloroplast genomes of five species of *Salvia* and performed phylogenetic analysis with current available CPs of *Salvia*. Repeated sequence analysis and comparative analysis of *Salvia* CPs were also performed with representative species from different distribution centers. The results showed that the genetic characters of the CPs are related to the geographic distribution of plants. Species from CAM diverged first to form a separate group, followed by species from EA, and finally species from CASA. Larger variations of CPs were observed in species from CAM, whereas more deficient sequences and less repeated sequences in the CPs were observed in species from CASA. These results provide valuable information on the development and utilization of the worldwide genetic resources of *Salvia*.

## Introduction

*Salvia* L. is the largest genus in the family of Lamiaceae, consisting of approximately 1000 species, distributed from the Far East through Europe to the New World, forming three broadly defined distribution centers of East Aisa (EA, ~ 100 spp.), Central and Western Aisa/Mediterranean (CAM, ~ 250 spp.), and Central and South America (CASA, ~ 500 spp.)^1–3^. Along with its large number of species and worldwide distribution, the genus is of great medicinal, horticultural and ecological importance^4^, widely used as medicinal materials, culinary herbs, as well as ornamental plants. On the basis of the morphological characteristics of the two aborted posterior stamens and their peculiar pollination mechanism, the genus *Salvia* was thought to be monophyletic apart from other genera in the mint family (Lamiaceae) in history. However, according to the molecular phylogenetic analysis with the chloroplast DNA regions *rbcL* and *trnL-F* conducted two decades ago, the infrageneric relationship within *Salvia* was proved to be polyphyletic^1^. Five previously recognized small genera (*Dorystaechas* Boiss. & Heldr., *Meriandra* Benth., *Perovskia* Kar., *Rosmarinus* L., and *Zhumeria* Rech.f. & Wendelbo) were proved to be nested within several clades of *Salvia*^2,5–11^. Subsequently, an expanded concept of *Salvia* with 11 subgenera (*Audibertia* Benth., *Calosphace* Benth., *Glutinaria* Raf., *Salvia* L., *Sclarea* Moench., ’Heterosphace’, *Dorystaechas* Boiss. & Heldr., *Meriandra* Benth., *Perovskia* Kar., *Rosmarinus* L., and *Zhumeria* Rech.f. & Wendelbo) was proposed^11,12^. Besides ‘Heterosphace’ which has a multi-distribution across the continents, *Audibertia* and *Calosphace* are distributed in CASA, *Glutinaria* in EA, and all other subgenera are distributed in CAM^12^.

Previous phylogenetic analyzes of *Salvia* were mostly based on DNA fragments from the nuclear genome or the chloroplast genome (CP). However, longer DNA sequences with more informative characters can improve the phylogenetic resolution and the support values of branches^13,14^. To overcome the inherent limitations and bias cost of the single or limited number of chosen loci, the complete CP containing more variation information is helpful to understand the relationships among basal angiosperms^15^. The complete CPs are efficiently used to increase phylogenetic resolution at lower taxonomic levels^16^ as they contained more than 100 times more simplified informative sites than the combination of only several DNA fragments^17^. CPs in angiosperms exhibit a typical conserved quadripartite structure, including a large single copy (LSC) region, a small single copy (SSC) region, and two inverted repeat (IR) regions^18^. The length of CP varies between 15,553 base pairs (bp) of *Asarum minus* and 521,168 bp of *Floydiella terrestris*^18^. The substitution rate for cpDNA genes was almost 10 times lower than that for genes from nuclear genomes^19^. CPs are uniparental inheritance (usually maternally inherited in most angiosperms), along with their stable genetic structure, leading to a low mutation rate during plant evolution. As a result, CP is a valuable tool for species identification on infrageneric level^20,21^ and for phylogenetic reconstruction^22^.

After the publication of the first CP of *Salvia* almost 10 years ago^23^, the number of CPs of *Salvia* uploaded to the GenBank has increased rapidly in the recent three years. In total, more than 100 CPs of *Salvia* have been published (with species repetition) so far, of which more than 90 were uploaded in the last three years (19 in the year 2020, 31 in the year 2021 and 42 in the year 2022) (Supplementary Table S1). The genomic characterization and phylogeny analysis of *Salvia* have been conducted with complete CPs two years ago with only 17^14^ or 19 species^17^, supporting the monophyly of *Salvia* and *Salvia* subg. *Glutinaria.* Such analyzes were conducted with species mostly originated from EA and CAM, and there was a lack of information of species originated from CASA. The genetic characters of CPs among the species originated from the three distribution centers were also not considered in previous studies. With more and more complete CP sequences available nowadays, it is possible to investigate the phylogeny of the genus *Salvia* and the variations among its three distribution centers with complete CPs.

In this study, we sequenced and assembled the CPs of 5 *Salvia* species (2 from EA and 3 from CASA). Besides, all available *Salvia* CPs were downloaded from GenBank for characterization. Phylogenetic analysis, repeated sequence analysis, and compare genome analysis were conducted with species from the three main distribution centers to understand the genetic features of CPs of each center. The differences and characters of the CPs of species originally from the three distribution centers were summarized.

## Results

### Features of chloroplast genomes in *Saliva*

In this study, the CPs of *Salvia azurea*, *S. iodantha*, *S. microphylla*, *S. nipponica*, and *S. umbratica* were sequenced. The GenBank accession numbers of these species are MT156371, MT156366, MT156365, MT156377, and MT156375, respectively. Additionally, all published CPs of species in *Saliva* were downloaded from the GenBank (up to June, 2023), and the characters of the CPs were listed in the Supplementary Material Table S1. The sizes of CPs *S. microphylla*, *S. iodantha*, *Salvia azurea*, *S. umbratica* and *S. nipponica* are 150,873 bp, 150,814 bp, 150,959 bp, 151,640 bp, and 151,484 bp, respectively (Table 1). These are very similar to other CPs of *Saliva* (Table S1). The lengths of the LSCs of these five species range from 82,102 bp to 82,870 bp (Table 1). The length of the LSC of *Salvia iodantha* (82,102 bp, Table 1) is the shortest among the published CPs in *Saliva*, while the longest LSC (84,573 bp) is from *Salvia japonica* (accession no. KY646163, Table S1). The lengths of the SSCs of the five species range from 17,432 bp to 17,627 bp (Table 1), which are within the range of lengths of SSCs in CPs of *Salvia*, from 16,974 bp to 17,962 (Table S1). The lengths of the IRs of the five species range from 25,584 bp to 25,601 bp (Table 1), which are within the range of lengths of IRs in CPs of *Salvia*, from 25,283 bp to 25,916 (Table S1). The GC content of all CPs in *Salvia* is around 38%, which is in consistent within *Salvia* (Tables 1, S1).

**Table 1:** Summary of the features of the chloroplast genomes of five *Salvia* species.

Species	Accession number	Distribution center	Genome size (bp)	LSC length (bp)	SSC length (bp)	IR length (bp)	GC content (%)	Number of genes			
								Total	Protein coding	tRNA	rRNA
*Salvia azurea*	MT156371	CASA	150,959	82,238	17,535	25,593	38.02	134	89	37	8
*Salvia iodantha*	MT156366	CASA	150,814	82,102	17,538	25,587	38	133	88	37	8
*Salvia microphylla*	MT156365	CASA	150,873	82,273	17,432	25,584	38.01	133	88	37	8
*Salvia nipponica*	MT156377	EA	151,484	82,747	17,627	25,555	37.95	134	88	37	8
*Salvia umbratica*	MT156375	EA	151,640	82,870	17,568	25,601	37.98	134	88	37	8

*CASA* Central and South America, *EA* East Asian.

**Figure 1:**
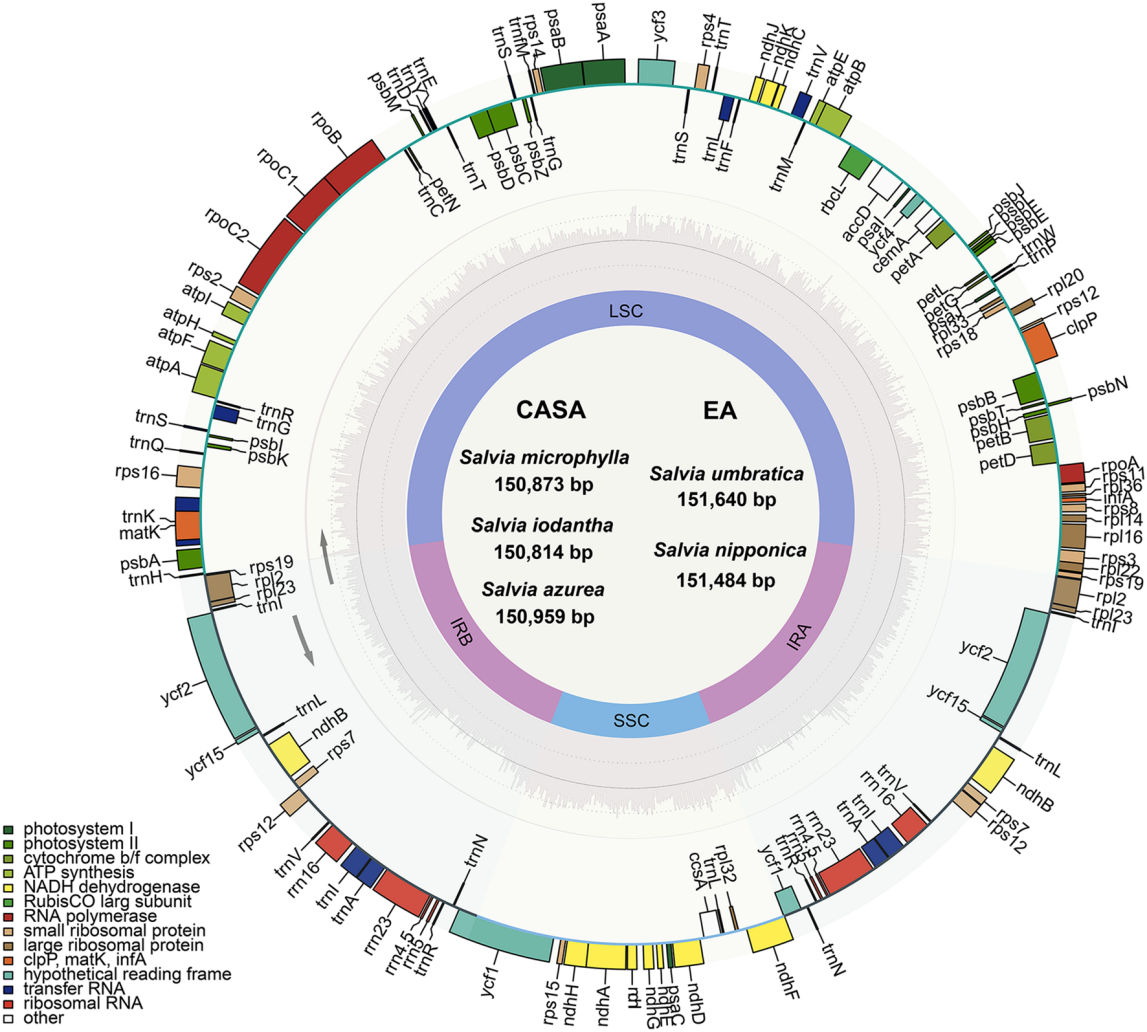
Gene map of *Salvia azurea*, *S. iodantha*, *S. microphylla*, *S. nipponica*, and *S. umbratica*. Genes that possess different functions are color coded. The inner gray circle represents the GC content. LSC, large single-copy region; SSC, small single-copy region; IR, inverted repeat. CASA: Central and South America; EA, East Asian.

The five sequenced CPs each contain 133 or 134 genes, including 88 to 89 protein coding genes (~ 65.2%), 37 tRNA genes (~ 27.7%) and 8 rRNA genes (~ 6.0%) (Table 1). The genes and their orders are almost the same among the five species (Fig. 1), and there are only tiny distinctions between the five species. The species of *Salvia azurea* (MT156371), *Salvia iodantha* (MT156366), and *Salvia microphylla* (MT156365) from CASA, each contains two *rps19* genes, whereas the species of *Salvia nipponica* (MT156377) and *Salvia umbratica* (MT156375) from EA, each contains only one *rps19* gene (Table S2), and thus there are one more gene in each of the three species from CASA than the two species from EA (Table 1). All these five species consist of two copies of a tRNA gene *trnN-GUU* in the IR region, but differentially from the other four species, the gene in the CP of *Salvia microphylla* (MT156365) contains an intron (Table S2).

### Phylogenetic analysis of *Saliva* based on the complete CPs

**Figure 2:**
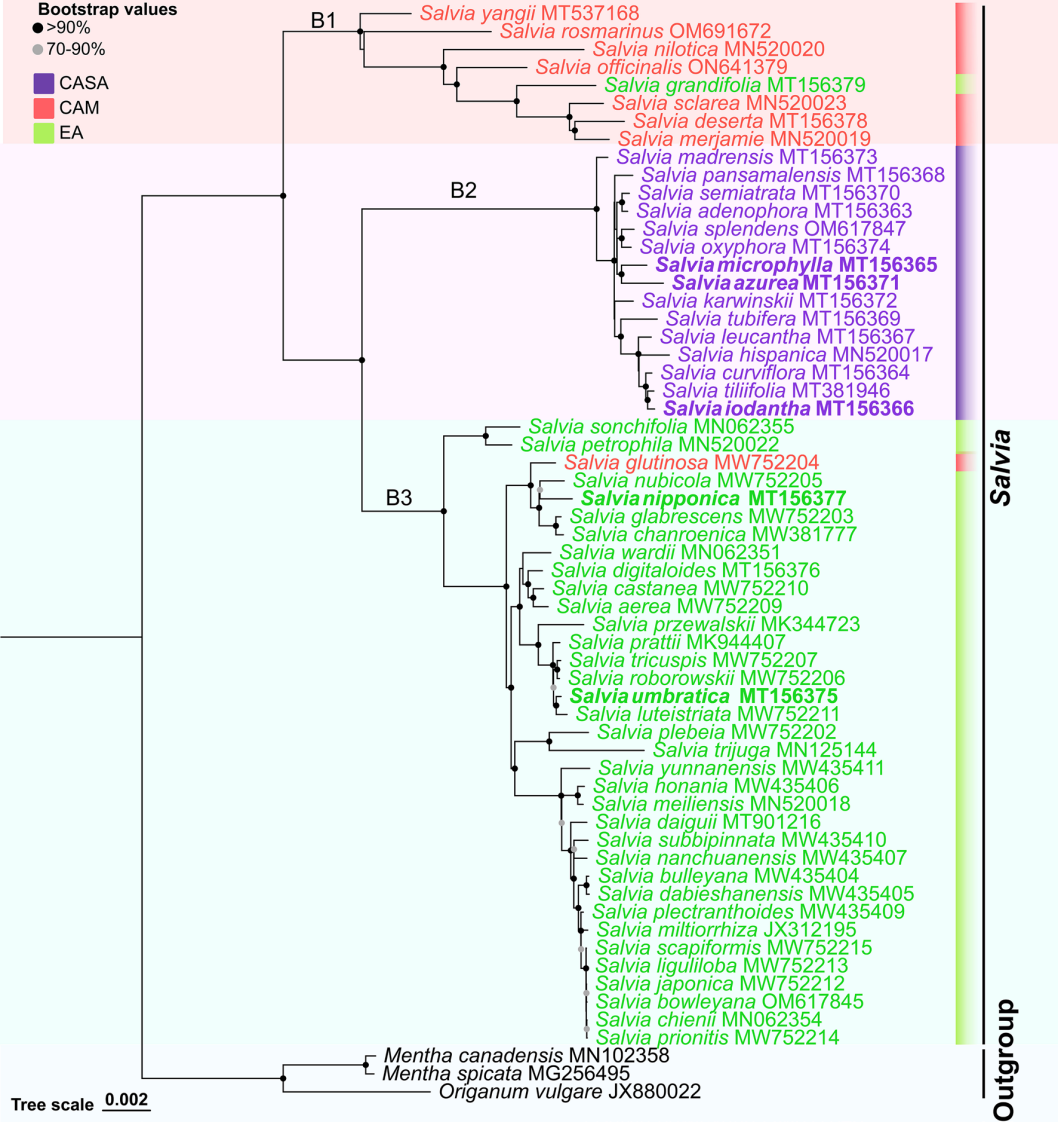
Phylogenetic tree constructed of complete chloroplast genomes currently published in *Salvia* based on the maximum likelihood method. Ranges of bootstrap values with 1000 replicates were shown by gray level at each node. CASA: Central and South America, CAM: Central Asia/Mediterranea, EA: East Asia, B1: the first main branch, B2: the second main branch, B3: the third main branch. Species marked as bold in the texts are those sequenced in this study.

Phylogenetic analysis was performed based on the maximum likelihood (ML) tree with current available complete CPs of 58 *Salvia* species (8 from CAM, 15 from CASA, and 35 from EA) and an outgroup of *Mentha canadensis*, *M. spicata,* and *Origanum vulgare*, species of the genus of *Mentha* of Lamiaceae. Species of *Salvia* were clearly separated from the outgroup and formed three main branches (Fig. 2). The first main branch (B1) was separated from the second and third main branches (B2 and B3), and then B2 and B3 were separated from each other (Fig. 2). The three main branches were generally consistent with the three distribution centers. Species from CAM were mostly clustered in B1 (Fig. 2), that were firstly separated from other species, indicating an early differentiation of these species. Within this branch, there was one species (*S. grandifolia*) from EA, showing that the branch was not formed with isolated species from CAM. B2 was a terminal branch with species all from CASA (Fig. 2), showing that the divergence time of species from CASA was much later than the other two centers. The CASA was geographically isolated from the CAM and EA, and correspondingly, all species from CASA formed a single branch and were suspected to be the newest in the evolution. B3 consisted of species mostly from EA, with one exception of *S.glutinosa* from CAM (Fig. 2). Thus, genetic communication across CAM and EA can be expected, considering that CAM and EA were actually continuous in the land of Eurasia. However, a closer genetic relationship between species in EA and CASA was suggested than to the species in CAM, since B3 and B2 were clustered prior to B1 (Fig. 2). In addition, according to the branch length of B3 (Fig. 2), the divergence time in EA was later than in CAM but earlier than that in CASA.

### Repetitive sequences in CPs of *Salvia* among three distribution centers

**Figure 3:**
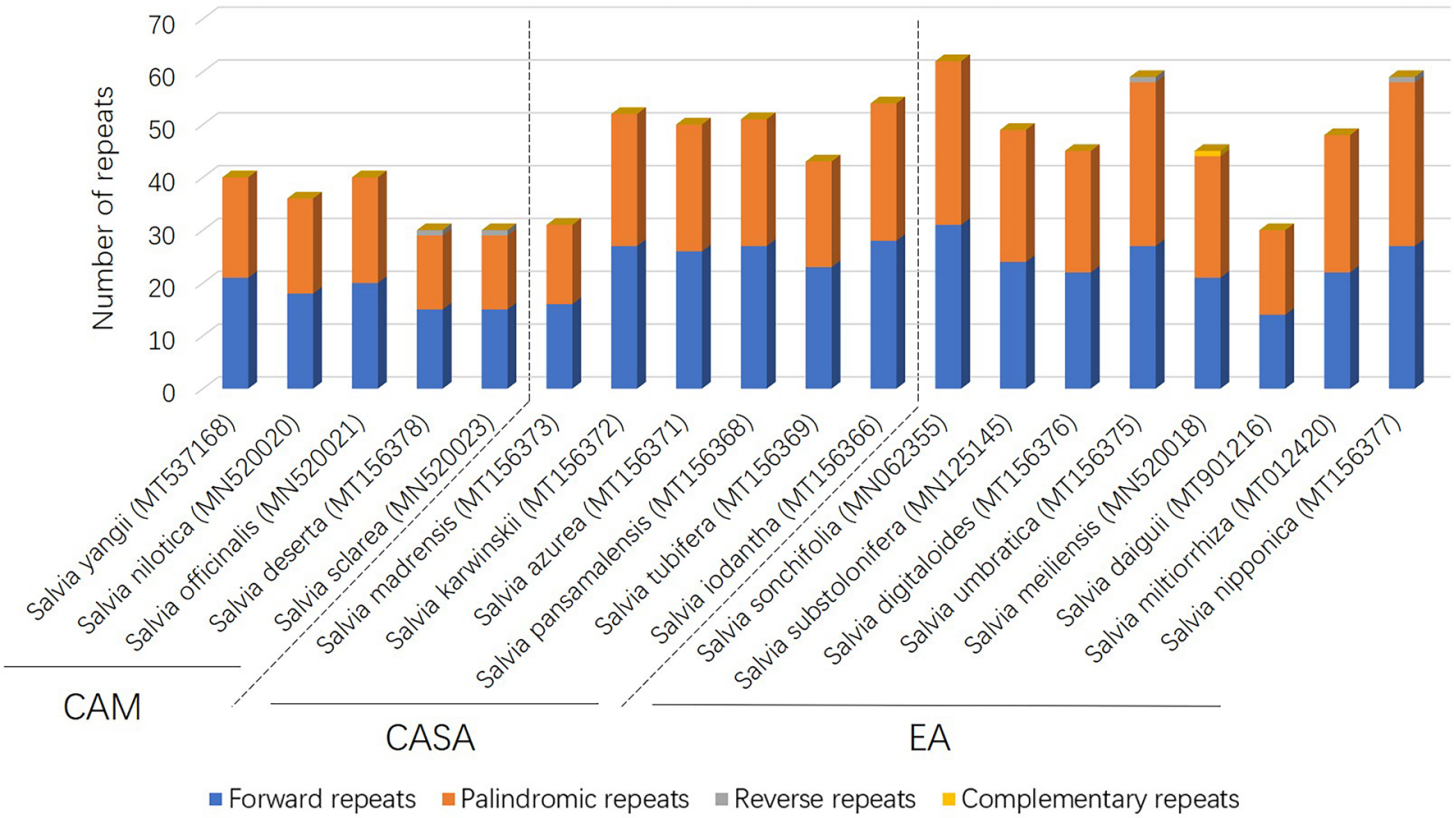
Interspersed repeated sequences analyses for CPs of *Salvia* species in the three main distribution centers.

Based on the phylogenetic analysis and the distribution region of the species, five to eight species (5 for CAM, 6 for CASA, 8 for EA) were chosen from each distribution center for repetitive sequence analyses. The number of long interspersed repeated sequences ranged from 30 to 62 among the 19 chosen species. Forward repeats and palindromic repeats were the major types, whereas reverse repeats and complementary repeats were rarely presented (Fig. 3). Among the three distribution centers, CAM has the least number of long repeats with an average of 32, and EA has the highest number of long repeats with an average of 50, whereas the number of long repeats of species in CASA is closer to that of EA, which was 47 as an average (Fig. 3). Interestingly, species in CAM and CASA have more forward repeats (or at least no less than) than palindromic repeats, whereas species from EA have more palindromic repeats than forward repeats (Fig. 3). Furthermore, two species in CAM and two species in EA each contains a reverse repeat, but no reverse repeat was identified in the species from CASA (Fig. 3). Species from CAM and CASA do not have complementary repeats, whereas one species from EA has one complementary repeat (Fig. 3).

**Figure 4:**
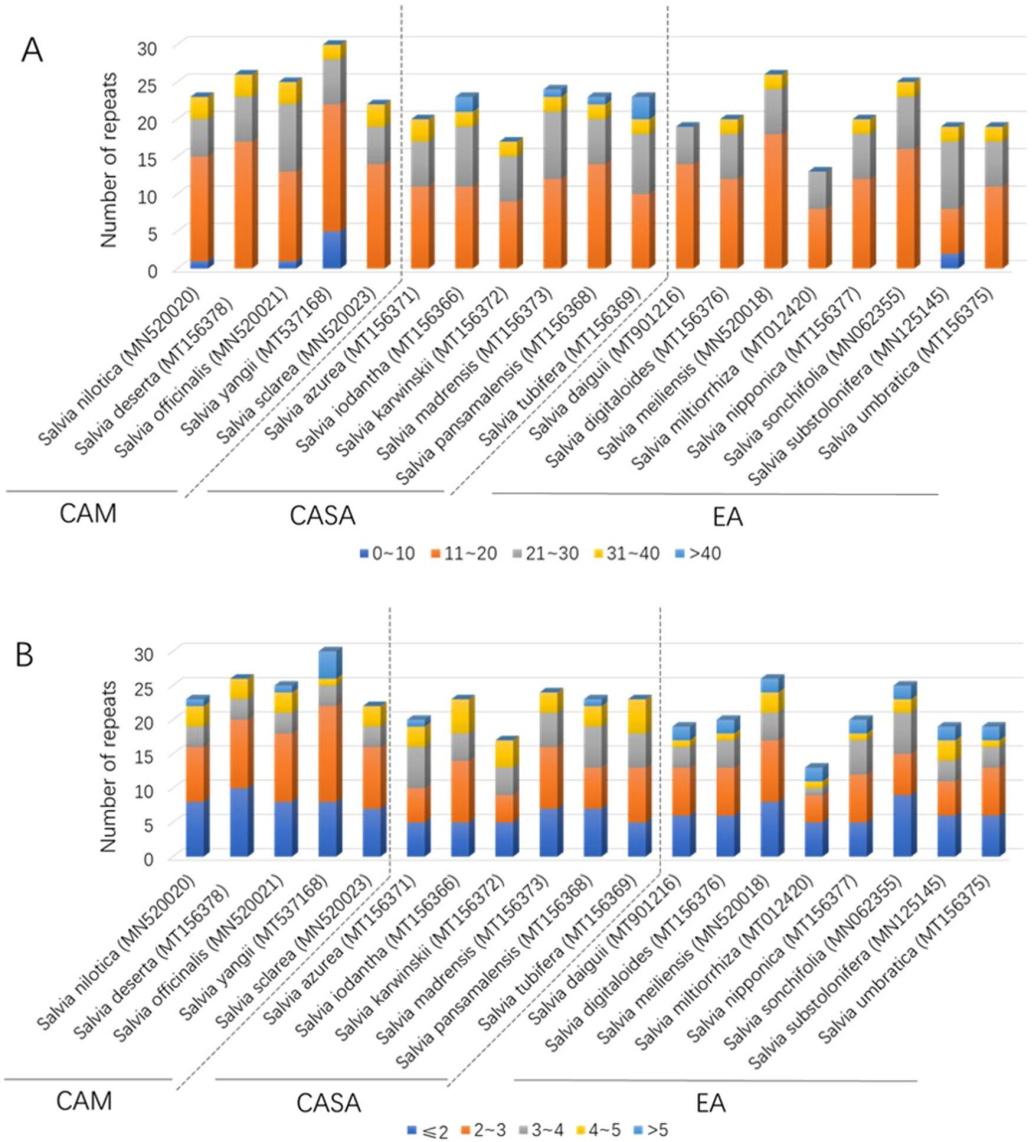
Tandem repeated sequences analyses for CPs of *Salvia* species in the three main distribution centers. A. Period size, B. Copy number.

The period size (repeat unit size) for tandem repeats of 19 species was mainly between 10 and 40 (Fig. 4A). Three species in CAM had tandem repeats with period size less than ten, and among them *S. yangii* had five such short tandem repeats, whereas, except one in EA, no species in CASA or EA had tandem repeats with period size less than ten (Fig. 4A). The major tandem repeats were those with period size between 10 and 20, with averages of 14.8, 11.2, and 12.1 of CAM, CASA and EA, respectively (Fig. 4A). The number of period size between 20 and 30 ranged from 5 to 9 and was evenly distributed in the three centers (Fig. 4). Interestingly, the long period size (larger than 40) existed only in the species in CASA, and the period size between 30 and 40 was more frequently in CAM and CASA (with 2 or 3 in each species) than in EA (with 2 or 0 in each species) (Fig. 4A). The copy number of most tandem repeats was less than 3, especially of the species in CAM (Fig. 4B). The frequency of copy number larger than 5 varied between 0 to 4 for CAM, 0 to 1 for CASA, but 2 for all eight species in EA (Fig. 4B).

**Figure 5:**
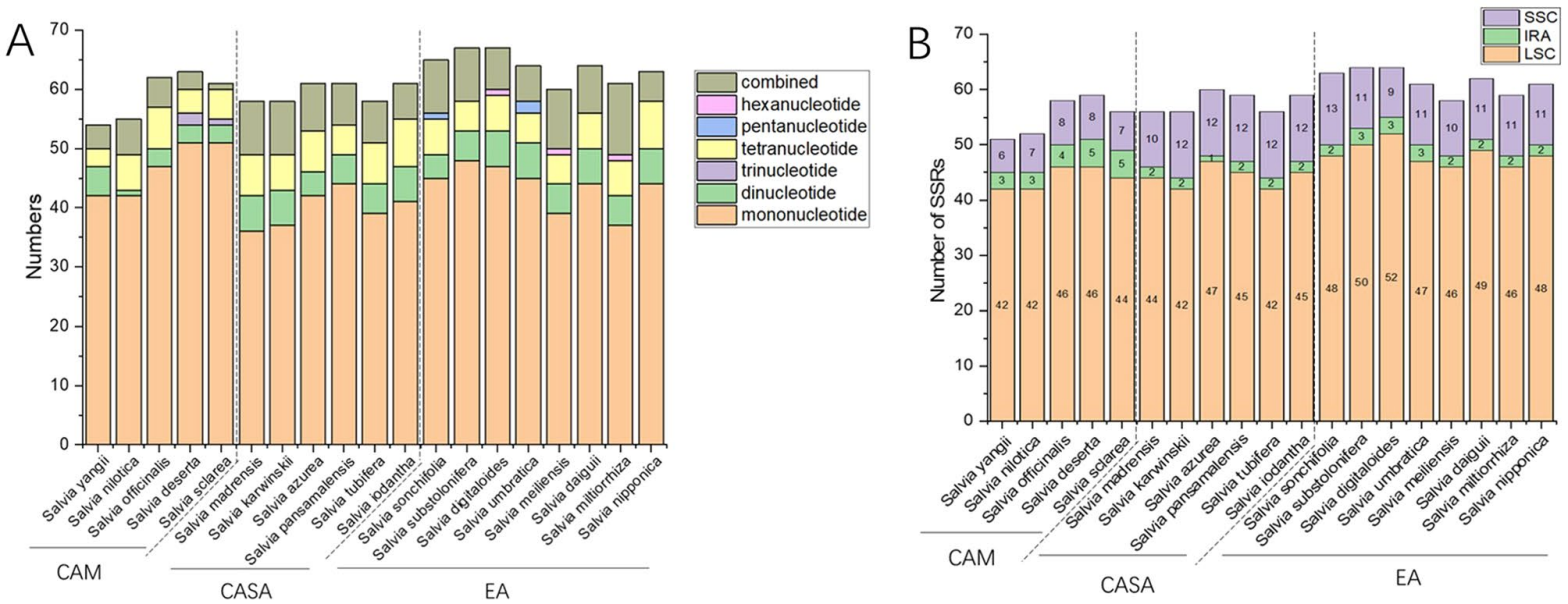
SSR analyses for CPs of *Salvia* species in the three main distribution centers. (**A**) Numbers of different types of SSR, (**B**) Numbers of SSR in each region of the CP.

The microsatellite, also named simple sequence repeats (SSR), is a special tandem repeat that usually acts as molecular markers for genetic analyses. In the genus of *Salvia*, the main type of SSR is mononucleotide, which was the most common, followed by combined type, tetranucleotide type, and dinucleotide type, while few tri-, penta- and hexan-nucleotide types were found (Fig. 5A). Most of the SSRs were located in the LSC region of the CP. There were ~ 44 SSRs in the LSC of species in CAM and CASA, and ~ 48 in the LSC of the species in EA (Fig. 5B). There were fewer SSRs in the SSC regions of species in CAM (~ 7) than in the SSC regions of species in CASA and EA (~ 11 to 12) (Fig. 5B). The number of SSRs in a single IR region is slightly higher in the species in CAM (~ 4) than in CASA and EA (~ 2) (Fig. 5B). As a result, the species in CAM and CASA contain fewer SSRs (~ 59) than the species in EA (~ 64) (Fig. 5B).

### Comparative genomic analyses among three distribution centers

**Figure 6:**
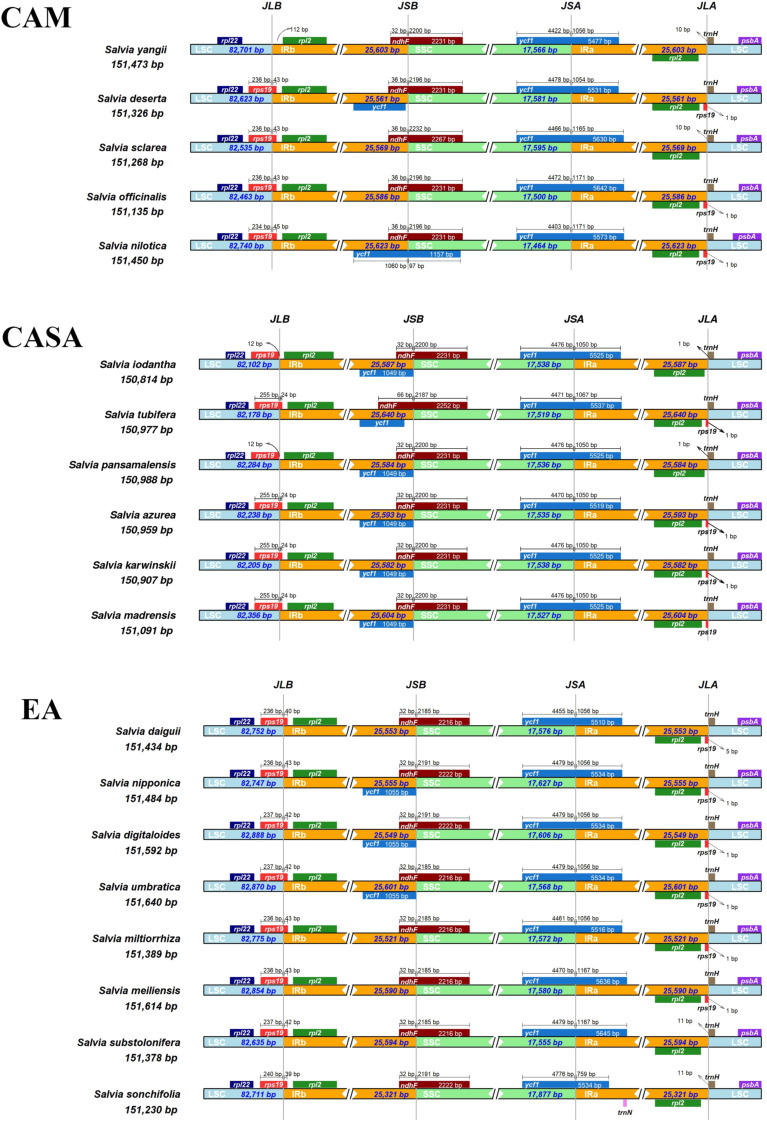
Comparison of junctions between the LSC, IR, and SSC regions among CPs of *Salvia* species in the three main distribution centers.

The adjacent genes and boundaries of the four regions of LSC-IRb-SSC-IRa of the species distributed in the three centers were compared (Fig. 6). Here, the boundary of IRb and LSC (JLB) was located within the coding region of the *rps19* gene, except for *S. yangii*. The *rps19* gene had an expansion of 43/45 bp in the IRb region of the species from CAM, 39 to 43 bp in the IRb region of the species from EA, whereas it did not expand to or only expanded for 24 bp in the IRb region of the species in CASA (Fig. 6). The *ndhF* gene is located at the boundary of IRb and SSC (JSB), which mostly contains about 32/36 bp in the IRb region and over 2000 bp in the SSC region (Fig. 6). The length of the *ndhF* gene varied between the species among three distribution centers. The length of the *ndhF* gene was 2231 in most cases in the species in CAM and CASA, whereas the length was 2216 or 2222 in the species in the EA (Fig. 6). The boundary of SSC and IRa (JSA) was located within the coding region of the gene *ycf1*, with an extension of 1050 or 1067 in SSC in the species in CASA, 1056 or 1167 in SSC in the species in EA, and 1054 to 1171 in the species in CAM (Fig. 6). This showed that the variation in the extension of *ycf1* in the IRa region of species in CAM covered the extension lengths of the *ycf1* gene in the IRa region of the species in CASA and EA.

**Figure 7:**
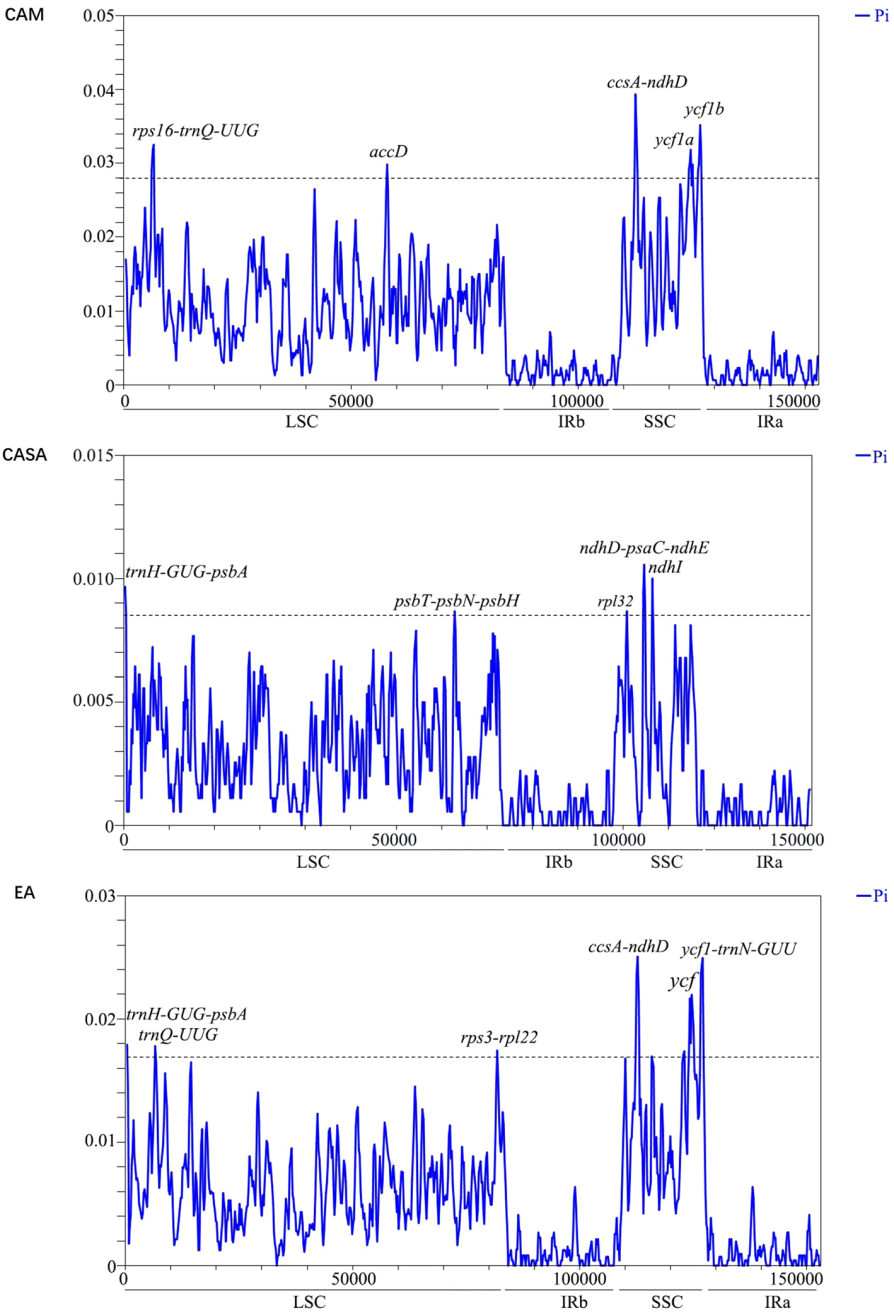
Sliding window analysis of CPs of *Salvia* among the three main distribution centers. Pi of 5, 6 and 8 plastomes of *Salvia* species from CAM, CASA and EA, respectively, are shown in the figures. Mutational hotspots and highly divergent loci are marked.

The nucleotide variability (Pi) values were calculated with the species from the three distribution centers for the sequence divergence analysis. The highly variable regions were mainly distributed in the LSC and SSC regions, but not in the IR regions (Fig. 7), showing the conservation of the IR regions. And the variable regions in the SSC region were generally higher than those in the LSC region in all three distribution centers (Fig. 7). The Pi values among the three centers showed great differences (Fig. 7). The ranges of the Pi values of the plastomes in the CAM, CASA, and EA were 0 to 0.0393, 0 to 0.0106 and 0 to 0.0251, respectively. This showed that the variation of the species in EA was two times higher than those in CASA and the variation of species in CAM was almost four times higher than those of CASA. These results confirmed the greater variation of CPs in CAM and EA than in CASA. The highly variable regions among the three centers were also very different (Fig. 7). In CAM, highly variable regions with Pi values > 0.028 were detected, including 3 genes (*accD*, *ycf1a*, and *ycf1b*) and 2 intergenic spacers (*rps16-trnQ-UUG* and *ccsA-ndhD*) (Fig. 7). In CASA, highly variable regions with Pi values > 0.0085 were detected, including 2 genes (*rpl32* and *ndh1*) and 3 intergenic spacers (*trnH-GUG-psbA*, *psbT-psbN-psbH* and *ndhD-psaC-ndhE*) (Fig. 7). In EA, highly variable regions with Pi values > 0.017 were detected, including 2 genes (*trnQ-UUG*, *ycf*) and 3 intergenic spacers (*trnH-GUG-psbA*, *rps3-rpl22*, *ccsS-ndhD*, *ycf1-trnN-GUU*) (Fig. 7). As a result, the intergenic spacer of *trnH-GUG-psbA* was common in EA and CASA, and the inter genic spacer of *ccsS-ndhD* was common in CAM and EA. Besides these, other highly variable regions were unique in each distribution center.

**Figure 8:**
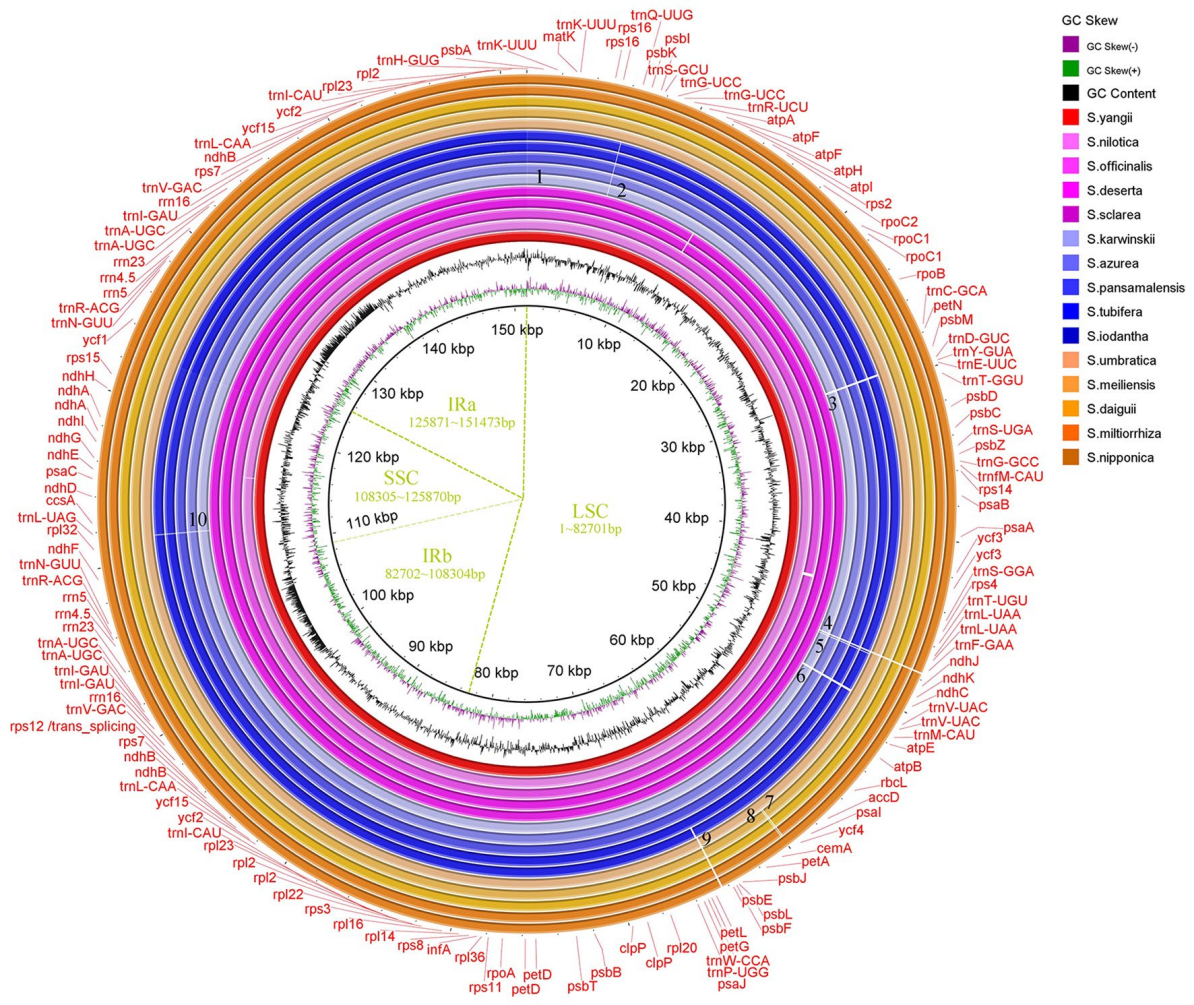
Comparison of the chloroplast genomes of *Salvia* species in the three main distribution centers. Circles from inner to outer represent CPs of species from CAM (*S. yangii*, *S. nilotica*, *S. officinalis*, *S. deserta* and *S. sclarea*, the red and pink circles), species from CASA (*S. karwinskii*, *S. azurea*, *S. pansamalensis*, *S. tubifera*, and *S. iodantha*, the blue circles) and species from EA (*S. umbratica*, *S. meiliensis*, *S. daiguii*, *S. miltiorrhiza*, and *S. nipponica*, the orange circles). The CP of *S.yangii* (the red circle) was used as reference for gene annotation. The black numbers on the colored circles marked the distribution-center specific missing regions of CPs of species compared to the reference.

The complete CPs of 15 species were compared from the three distribution centers (5 of each) (Fig. 8). With the species *S. yangii* from CAM, which was the oldest species according to the phylogenetic analysis (Fig. 2), as reference, it is clear to see the center-specific CP characters. Ten missing regions (longer than 20 bp) representing each distribution center were observed in the CPs, among which nine were located in the LSC region and one in the SSC region (Fig. 8). All missing regions were located in intergenic regions. The 1st, 2nd, 3rd, 5th, 6th and 10th missing regions were specifically observed in CASA, whereas the 9th missing region was only observed in species in EA (Fig. 8). The 7th and 8th missing regions were also EA-specific, but were only observed in three out of five species used in the analysis (Fig. 8). In addition, the 4th missing region was observed in both CASA and EA compared to CAM (Fig. 8). This observation was consistent with the phylogenetic analysis, that species in EA were closer to the species in CAM and the species from CASA were the latest split from the species in other regions.

## Discussion

The average length of current available CPs of *Salvia* is 151,370 bp, which is in consist with the average length of CPs of sequenced land plants (151 kb)^24^. Based on the average genome sizes of species from each distribution center, species from EA have the longest CPs (151,550 bp), slightly longer than the CPs of the species from CAM (151,325 bp), and the species from CASA have the shortest CPs (151,074 bp). Like most CPs of plants, CPs of *Salvia* display a typical quadripartite structure of small single-copy (SSC), large single-copy (LSC) and two copies of inverted repeat (IR) regions^25^. The CPs of *Salvia* are conserved within the genus and among the three distribution centers. The average number of genes of CPs of species in the three distribution centers are identical, which is 133 genes in total, including 88 protein coding genes in the CDS region, 37 tRNA genes and 8 rRNA genes. The average GC content is also identical among the three distribution centers, which is 38% of the whole genome, 36% in the LSC region, 32% in the SSC region and 43% in the IR region. These characteristics were consistent with the previously reported *Salvia* CPs^14,23,26–28^. In addition, the genes and their orders are also conserved among different centers (Fig. 1). The genes with variation in numbers are all protein coding genes, and the number of tRNA genes and rRNA genes were more conserved in the CPs of all *Salvia* plants (Table S1). All of these indicated the conservation of the CP features of all *Salvia* species.

Based on the phylogenetic analysis conducted by the complete genome of CPs, the three main branches corresponding well to the geographical distributions of *Salvia* plants (Fig. 2). Several species from CAM were clustered and formed B1 as the oldest branch, and then all species from CASA and almost all species from EA were clustered into two single branches (B2 and B3), respectively (Fig. 2). All species from CASA in this study were clustered in one main branch (B2, Fig. 2), suggesting that these species are monophyletic. They all belong to the subgenus *Calosphace*, which covers about half of the species of *Salvia* and almost all native *Salvia* species in America^29^. This is consistent with the isolation of the continent of America. All species from EA are also supposed to be monophyletic and belong to the subgenus *Glutinaria*^30^*.* However, the special one in this study, *S. grandifolia*, is an exception, belonging to the subgenus *Sclarea*^31^. *S. grandifolia* was clustered with other species of *Sclarea* from CAM in B1 (Fig. 2). In addition, *S.glutinosa*, a species from CAM, was clustered with species in EA (Fig. 2). Thus, the communication of DNA fragments in CPs can be expected between species from CAM and EA. Iran, with 60 species of *Salvia*, located in the CAM near to EA, is a center of origin for genus *Salvia*^32^, and Southwest Asia is estimated to be the location where diversification events of *Salvia* took place^12^. In this study, *S. yangii* in CAM was supposed to be the oldest species according to the phylogenetic analysis (Fig. 2). The native range of *S. yangii* is from Afghanistan to West China (Table S1), supporting that the origin of *Salvia* is the Southwest Aisa. Based on the results of the phylogenetic analysis (Fig. 2), though with only eight species from CAM, the divergence time of species from CAM varied much longer than species from the other two distribution centers. This indicated a big variation among species in CAM. The divergence time of species from EA was completely within the divergence time of species from CAM. The divergence time of all species was very close to each other and was much later than that of the species in CAM and EA, supporting that they are the newest species in the genus of *Salvia*^12^. Thus, the phylogeny analysis with complete CPs potentially confirms the previous suggested geographical expansion of *Salvia*, which is originally from western Asia to a nearly cosmopolitan distribution, first to eastern Asia and Europe and then terminated in the America (the New World)^1,6^.

In this study, variations on the numbers and types of repetitive sequences in CPs have been identified among the species from three distribution centers. Repetitive sequences in CPs were essential to understand the evolution of CP and genome rearrangements^33^. In this study, we found that forward repeats and palindromic repeats are the two main types of long interspersed repetitive sequences (> 30 bp) in the genus *Salvia*, which has also been reported by Su et al.^28^. Interspersed repeated sequences are the predominant type of repeats in the genome, and they are responsible for the expansion of the plastome and the variation of genome sizes among species^34,35^. However, in this study, species from CAM with the medial length of CPs have fewer interspersed repeats than the species from EA (with longer CPs) or CASA (with shorter CPs), suggesting that later differentiated species tend to have more interspersed repeats. Tandem repeats are adjacent repetitive DNA in the genome, which are instable, and therefore evolutionarily pertinent^34^. The addition or deletion of repeat units usually leads to polymorphisms of the tandem repeats. CPs of species from CAM include more short tandem repeats, whereas CPs of species from CASA include more long tandem repeats. This indicated that the repeat units of tandem repeats were accumulated during the dispersal of *Salvia* species from CAM to CASA. Due to the polymorphisms of the tandem repeats, they have been widely used as genetic markers in genetic diversity and plant population studies for a long time, especially SSR, a special type of tandem repeats^36,37^. There are more SSRs in the LSC region than in the SSC or IR region, indicating a higher variable in sequences of the LSC region. These results are similar to most of the SSRs of the CPs, as well as in other species^33,38–40^. Interestingly, though the total number of SSRs in the CPs of *Salvia* species from three distribution centers is similar, CPs of species from CAM contain more mononucleotide repeats compared to CPs of species from the other two distribution centers. This indicated a longer evolution time for the species from CAM.

It is generally believed that the variable boundary drives the force for the variation in CPs^41^. In the plastome of *Paphiopedilum*, the LSC/IR boundary is quite stable, but the SSC/IR region show variation^24^. In Magnoliidae, the changes in IR regions are related to the length of the gene *rps19*^42^. Within the species in *Salvia*, we did not find drastic expansions or contractions among different distribution centers. But there are specific changes at the LSC/IR boundary between different species related to the gene *rps19*. In the five CP sequences reported in this study, two *rps19* genes were identified in the species from CASA (*Salvia azurea*, *Salvia iodantha,* and *Salvia microphylla*), but only one in the species from EA (*Salvia nipponica* and *Salvia umbratica*) (Table S2). However, the number of *rps19* is not geographically specific. Almost all CPs contain a *rps19* at the LSC/IR boundary, but only some contain another *rps19* at the SSC/IR boundary, and this is not related to the three distribution centers (Fig. 6). Additionally, the gene *rps19* located in the LSC/IR boundary expand to IR region for 40 bp in the species from CAM and EA, but only 24 bp or no expansion in the species from CASA (Fig. 6). This indicates the variation of the IR regions of *Salvia* CPs among the three distribution centers. Since the species in CASA are last differentiated from the other two distribution centers, it is suggested that IR regions from CPs of the later formed species tend to be mildly contracted than the earlier formed species in *Slavia*.

Based on the comparison of the complete CPs from three distribution centers (represented by 5 species from each), the genomes are conserved in each of the distribution (Fig. 8). With the CP of the oldest species *S. yangii* originated from CAM in this study as reference, it is clear that many genome deletions occurred in the species from EA and CASA during the spread and evolution of *Salvia*. All deletion regions are between the genes, showing the conservation of the CP functions. There are common deletions between EA and CASA, but most deletions are observed only in CASA. This indicates that during the evolution of *Salvia*, more intergenic sequences of CPs are missing in later divergent species. This is also supported by the shortened length of the CPs of species from CASA, compared to that of the species from CAM or EA. The formation of the unique characters of CPs of species in CASA may be related to its geographic isolation. Interestingly, there are far more species of *Salvia* from CASA than the other two distribution centers. However, the development and utilization of *Salvia* species in CASA is far behind those of the species from CAM and EA. The percentage of species whose CP have been sequenced in each center is also unbalanced, as there are about 3.2% in CAM (8 out of ~ 250), 41% in EA (41 out of ~ 100), and about 3% in CASA (15 out of ~ 500). CPs of nearly half of the species in EA have been sequenced, but very rare CPs of species in CAM and CASA have been reported, though there are much more species in CAM and especially CASA compared to EA. In addition, the CP of *Salvia officinalis* from CAM itself has been sequenced 8 times to date, but the CPs of species from CASA have rarely been sequenced more than 2 times (Table S1). *Salvia* species, such as *Salvia officinalis* and *Salvia miltiorrhiza* in CAM and EA, have been used as medicines with a long history. The long-term utilization of species provokes the studies of certain species and even expanded to its substitution recourses^43^. Comparing to the large number of species in the genus of *Salvia* in total, only about 10% of species whose CPs have been reported. Therefore, to provide more information on the development and utilization of plant resources of the genus *Salvia* and to fully understand the genetic relationship and evolution of the genus, much more information and analyses of complete CPs are still needed in the future, especially those of the species from CASA.

## Materials and methods

### Sample collection, DNA extraction and sequencing

The plant materials of *S. azurea*, *S. microphylla*, *S. iodantha*, *S. umbratica* and *S. nipponica* were collected from the Chiapas Madre Mountains (Chiapas, Mexico), Yuma County (Arizona, USA), Monterrey (Nuevo Leon, Mexico), Mentougou District (Beijing, China), Taipei (Taiwan, China), respectively, and each was identified by DNA barcoding technology based on ITS2 markers. The specimens were deposited in the herbarium of the Institute of Chinese Materia Medica, China Academy of Chinese Medicinal Sciences, Beijing, China under the voucher number SZ20190620, SP20190023, SH20190025, SY20190112, SD20190201. Fresh and healthy leaves were collected from each individual plant of each species and were immediately frozen in liquid nitrogen prior to DNA extraction. The total genomic DNA was extracted using a modified cetyltrimethylammonium bromide (CTAB) method^44^ for DNA genome sequencing. Paired-end (150 bp) sequencing was performed on the Hiseq 1500 platform (Illumina Inc., USA).

### Chloroplast genome assembly and annotation

The sequence of the complete chloroplast genome of *S. miltiorrhiza* (NCBI accession number: NC020431) was used as the reference genome for extracting chloroplast genome reads^23^. The complete chloroplast genomes were assembled by SOAPdenovo (version 2.04)^45^. The complete chloroplast genome of *S. azurea*, *S. microphylla*, *S. iodantha*, *S. umbratica* and *S. nipponica* were submitted to GenBank after being annotated by Plann^46^ with NCBI accession number MT156371, MT156365, MT156366, MT156375, and MT167377, respectively.

Features of the five sequenced *Salvia* chloroplast genomes and published *Salvia* chloroplast genomes (up to June, 2023) were summarized in Supplemental Table S1. The accession number, upload data, and number of protein coding genes of each CP sequence were searched from NCBI. The distribution center (CAM, CASA, or EA) for each species was determined according to the information from Kew Backbone Distributions (https://powo.science.kew.org/). The lengths of SSC, LSC and IR regions were calculated after the boundaries of these regions obtained through Blastn software (version 2.6.0)^47^. The GC contents of each whole chloroplast genome, LSC, SSC, and IR regions were determined through Extractseq software (version 6.6.0)^48^ and Seqkit software (version 2.3.1)^49^. The number of genes for the total chloroplast genome, tRNA, and rRNA were determined from the .gb file of each chloroplast genome.

The annotations of the five sequenced *Salvia* chloroplast genomes were checked using CpGAVAS^50^. Gene names and their categories, groups, and numbers in the five newly assembled chloroplast genomes were summarized and listed in Supplemental Table S2. The gene map of the five newly assembled *Salvia* complete CPs was drawn using the online tool IRscope (https://irscope.shinyapps.io/Chloroplot/).

### Phylogenetic analysis

Five newly assembled *Salvia* complete chloroplast genomes and other chloroplast genomes of *Salvia* downloaded from NCBI (after removing the questionable sequences, only one was included for the duplication species), representing current available CPs of *Salvia*, were used for phylogenetic analysis (details see Supplemental Table S1), together with *Mentha canadensis*, *M. spicata*, and *Origanum vulgare* as an outgroup. MAFFT (version 7.310)^51^ was used to complete the alignment, and then the maximum likelihood (ML) tree was performed using RAxML (version 8.2.12) with the GTRGAMMA model and 1000 ultrafast bootstrap replicates^52^.

### Repeated sequence analysis, sequence divergence analysis, and comparative analysis of* Salvia* chloroplast genomes in the three distribution centers

Five, six, and eight species from CAM, CASA, and EA, respectively, were chosen from each distribution centers for further analyses. Long repeats of each species were detected by using the online tool REPuter^53^. The hamming distance was 3, and the minimum sequence length was 30 bp, and four match directions (F: forward, R: reverse, C: complementary and P: palindromic) were chosen. Microsatellite (SSR) analysis was carried out using the online MIcroSAtellite (MISA) identification tool^54^. The parameters were set as follows: nine repeat units for mononucleotide SSRs, five repeat units for dinucleotide SSRs, four repeat units for trinucleotide SSRs, and three repeat units for tetra-, penta- and hexanucleotide SSRs. The minimum distance between two SSRs was set to 100 bp.

The sequences of chosen CPs from each distribution center were aligned using Geneious Prime v2021.1^55^. Sliding window analysis was performed to generate nucleotide diversity (Pi) of the chloroplast genomes using DnaSP (DNA Sequences Poly-morphism version 6.12.03) software^56^. The step size was set to be 200 bp, with a 600 bp window length. The threshold of Pi for highly variable regions of CAM, CASA and EA were set to be 0.025, 0.0085, and 0.017, respectively.

IR expansion and contraction in the chloroplast genomes of the representative species of *Salvia* species from EA, CAM, and CASA regions, respectively, were detected using IRscope in the online website https://irscope.shinyapps.io/irapp/. The visualization of the comparison of chloroplast genomes were performed using BRIG^57^.

### Ethical approval and consent to participate

The plant specimen is not designated as endangered species. It requires no specific permissions or licenses. Collection was conducted in accordance with guidelines provided by the Institute of Chinese Materia Medica, China Academy of Chinese Medical Sciences and national and international regulations.

### Supplementary Information

Supplementary Table S1.

Supplementary Table S2.

## Data Availability

All data generated or analyzed during this study are included in this published article and its supplementary information files. The raw sequence data reported in this paper have been deposited in the Genome Sequence Archive (Genomics, Proteomics & Bioinformatics 2021) in the National Genomics Data Center (Nucleic Acids Res 2022), China National Center for Bioinformation/Beijing Institute of Genomics, Chinese Academy of Sciences (GSA: CRA010089) that are publicly accessible at https://ngdc.cncb.ac.cn/gsa. The assigned accession of the submission is CRA010089, and the associated BioProject is PRJCA015371 at https://ngdc.cncb.ac.cn/search/?dbId=bioproject&q=PRJCA015371.
